# Supplementary material for: Reversible and spatiotemporal control of colloidal structure formation
Source: Nat Commun. 2021 Nov 23;12:6811. doi: 10.1038/s41467-021-27016-x (PMC8611085; doi:10.1038/s41467-021-27016-x)
Supplement: Supplementary file 1 — Supplementary Information [file 41467_2021_27016_MOESM1_ESM.pdf]

# Supplementary Information

## Reversible and spatio-temporal control of colloidal structure formation

Dehne, H., Reitenbach, A. and A.R. Bausch

### Design of the DNA sequences

All oligonucleotides were purchased from *biomers* with HPLC purification and were designed based on the PEN-toolbox (molecular predator-prey system[31]) and checked using NUPACK. The autocatalytic DNA amplification of δ and ε is realized by the repetitive DNA sequences of $\overline{\delta\text{to}\delta}$ and $\overline{\varepsilon\text{to}\varepsilon}$. In total, a primer strand induces the amplification of additional primer strands without being consumed, effectively enhancing the reaction speed. The negative feedback mechanism, which regulates the amplification of δ is based on the palindromic sequence of η. Here, δ binds η, gets elongated by the polymerase and is thus transformed into a second strand η, which leads to autocatalytic consumption of the δ pool. The template strands were modified with a phosphorothioate backbone to prevent the degradation of the exonuclease and a 3' Phosphate was used to prevent the unintended elongation of the strands by the polymerase. In addition, Biotin modifications were used to realize the functionalisation of the streptavidin coated microspheres.

| **Name** | **Sequence (5'→3')** | **Modification** |
|---|---|---|
| $\delta$ | CATTCGGCCG | |
| $\overline{\delta\text{to}\delta}$ | C*G*G*CCGAATGCGGCCGAATG | 3'-Phosphat |
| $\varepsilon$ | CATTCAGACG | |
| $\overline{\varepsilon\text{to}\varepsilon}$ | C*G*T*CTGAATGCGTCTGAATG | 3'-Phosphat |
| $\eta$ | CATTCGGCCGAATG | |
| $\varepsilon$ poly t | A*A*A*ACGTCTGAATG | 3'-Phosphat |
| $\overline{\varepsilon\text{to}\delta}$ | C*G*G*CCGAATGCGTCTGAATG | 3'-Phosphat |
| $\overline{\delta\text{to}\varepsilon}$ | C*G*T*CTGAATGCGGCCGAATG | 3'-Phosphat |
| $\overline{\alpha}$ | C*C*C*ACCCGAATGGCATTACTTTCCGTCCCGAGAG<br>ACCTAACTGACACGCTTCCCATCGCTA | 3'-Biotin |
| $\overline{\beta}$ | AGCATTACTTTCCGTCCCGAGAGACCTAACTGACAC<br>GCTTCCCATCGCTAXTTACTATATAAC | 5'Biotin, 3'-Phosphat<br>X=C18spacer |
| $\overline{\gamma}$ | AGCATTACTTTCCGTCCCGAGAGACCTAACTGACAC<br>GCTTCCCATCGCTAXTCTTCTTCTTCC | 5'-Biotin, 3'-Phosphat<br>X=C18spacer |
| Spacer | T*A*G*CGATGGGAAGCGTGTCAGTTAGGTCTCTCG<br>GGACGGAAAGTAATGC | 3'-Biotin |
| $\overline{\delta\text{to}\alpha\beta}$ | T*T*A*CTATATAACCCCACCCGAATGCGGCCGAATG | 3'-Phosphat |
| $\overline{\varepsilon\text{to}\alpha\beta}$ | T*T*A*CTATATAACCCCACCCGAATGCGTCTGAATG | 3'-Phosphat |
| $\overline{\delta\text{to}\alpha\gamma}$ | T*C*T*TCTTCTTCCCCCACCCGAATGCGGCCGAATG | 3'-Phosphat |
| $\overline{\varepsilon\text{to}\alpha\gamma}$ | T*C*T*TCTTCTTCCCCCACCCGAATGCGTCTGAATG | 3'-Phosphat |
| $\alpha\beta$ | CATTCGGGTGGGGTTATATAGTAA | |
| $\alpha\gamma$ | CATTCGGGTGGGGGAAGAAGAAGA | |

| | | |
|---|---|---|
| $P_{\overline{\delta to \alpha\beta}}$ | T*T*A*CTATATAACCCCACCCGAATGCGGCCGAATG GCATTACTTTCCGTCCCGAGAGACCTAACTGAC ACGCTTCCCATCG | 3'-Biotin |
| $P_{\overline{\varepsilon to \delta}}$ | C*G*G*CCGAATGCGTCTGAATGGCATTACTTTCCGT CCCGAGAGACCTAACTGACACGCTTCCCATCGC TA | 3'-Biotin |

**Supplementary Table 1: DNA sequences used in this study.** Phosphorothioate backbones (*) are used to block the exonuclease digestion and the Phosphate at the 3' end of the DNA to prevent the elongation of the strands by the polymerase. The C18 spacer of the Docking-sequences prevents the elongation of the bound linker.

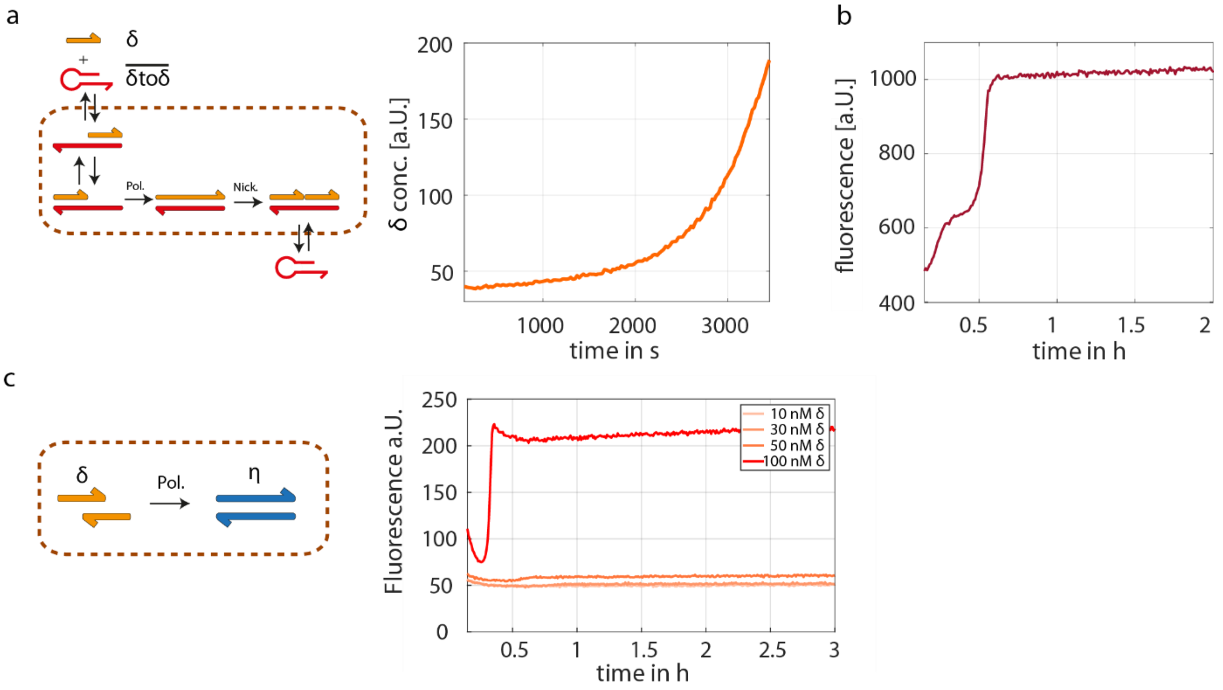


**Supplementary Fig. 1. Amplification of $\overline{\delta to \delta}$. a,** Scheme of the autocatalytic $\overline{\delta to \delta}$ reaction and the corresponding fluorescence signal monitored using *EvaGreen* ($\overline{\delta to \delta} = 120nM, \delta = 10nM$). The template $\overline{\delta to \delta}$ forms a DNA hairpin with a short overhang, which is opened by the binding of the δ strand. The δ strand acts as a primer and is elongated by the Bst DNA polymerase along the 5'→3' direction. The double stranded complex contains a recognition sequence for the Nickase Nb.Bsmi, which enables the production of two δ strands, because of the repetitive sequence of $\overline{\delta to \delta}$. **b,** The exponential growth of the δ strand ends in a stationary plateau due to the transformation of δ into η (here, $\overline{\delta to \delta} = 400nM$). **c,** Scheme of the spontaneous δ to η transformation. The δ strands have a short complementary sequence of 5nT, which leads to a weak interaction and the formation of an unstable double stranded complex at high concentrations. A series of δ concentrations were measured over time in the absence of $\overline{\delta to \delta}$ strands. The δ concentrations between 10 - 50 nM stay constant over the entire experiment, while higher concentrations (100 nM) result in a fast increase of the signal due to the transformation into η strands.

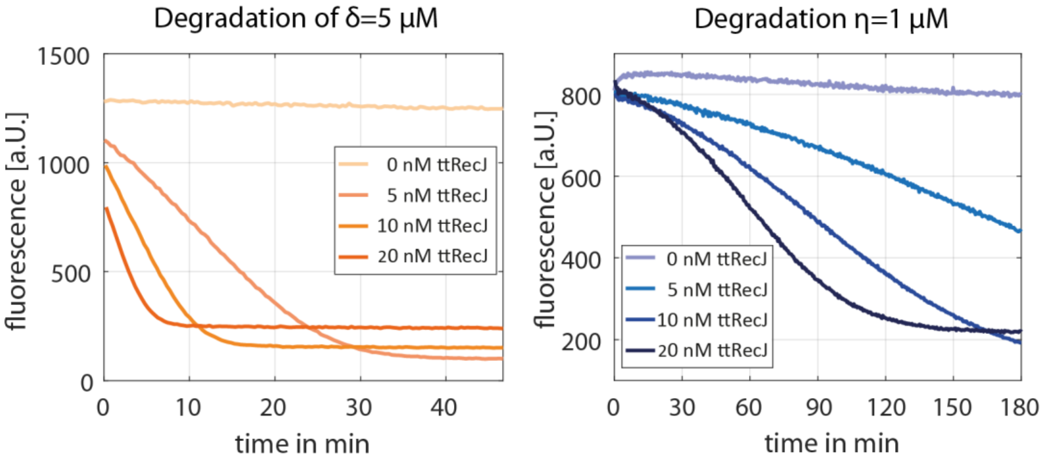


**Supplementary Fig. 2. Degradation of δ and η.** The degradation of δ and η was measured for an identical series of ttRecJ exonuclease concentrations. The degradation of 5 µM δ is completed in 30 minutes for the lowest ttRecJ concentration, while the degradation speed of η is significantly lower for the same enzymatic conditions. The reduction of the ttRecJ activity towards η is due to the preferred double stranded conformation of η strands and the single stranded degradation of the exonuclease (5'->3' exonuclease activity).

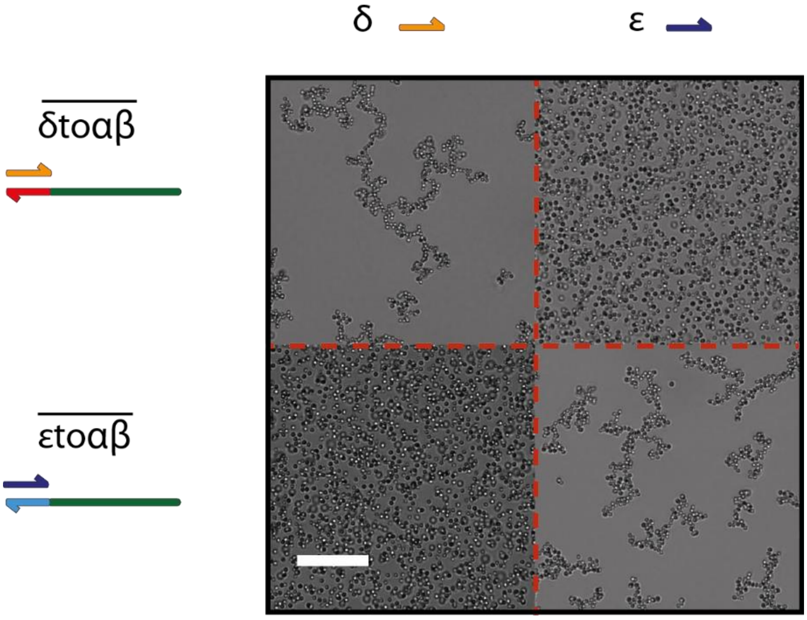


**Supplementary Fig. 3. Selectivity of the linker production.** The linker production of $\overline{\delta to\alpha\beta}$ and $\overline{\varepsilon to\alpha\beta}$ (50nM) is exclusively activated by the corresponding primer δ and ε (20nM) and results in a selective colloidal structure formation (scalebar=20µm, t= 60 min).

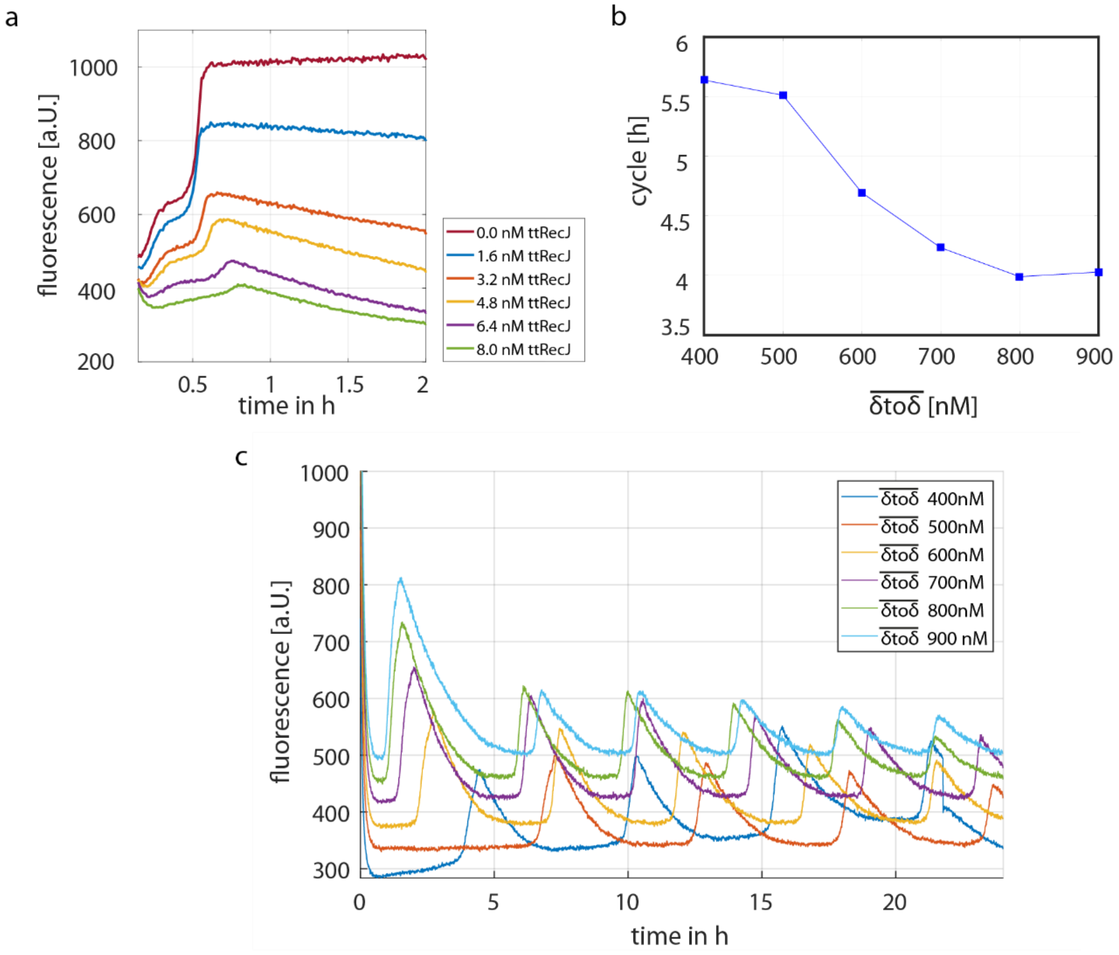


**Supplementary Fig. 4. Molecular predator prey system. a,** The δ amplification is monitored at $\overline{\delta to \delta}$=400 nM for increasing ttRecJ concentrations in the absence of η strands. The sample without exonuclease shows a two-step growth process. Here, the autocatalytic δ amplification is followed by the spontaneous η transformation, which finally results in a stationary phase. The peaks of the pulses decrease with the ttRecJ concentration and the duration of the decay can be controlled. **b,** Frequency shift for different concentrations of $\overline{\delta to \delta}$ (shown in Fig. 4c). **c,** Oscillations were monitored for different $\overline{\delta to \delta}$ concentrations. At 400 nM of templates, sustained oscillations can be obtained, while the increase of the template concentration results in the occurrence of damped oscillations. Here, an increased frequency can be observed, which is due to a reduction of the lag-phase between two pulses.

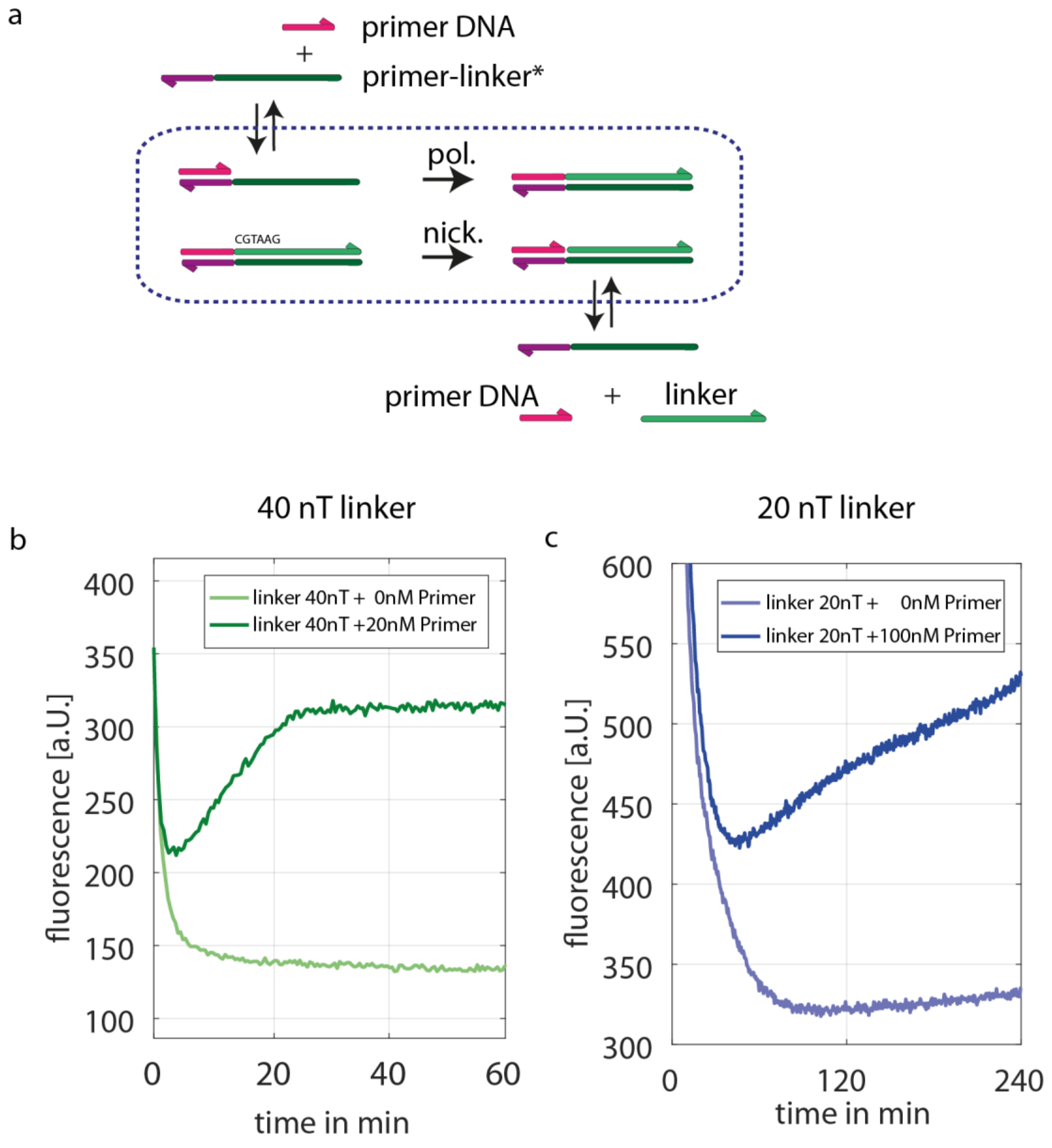


**Supplementary Fig. 5. DNA linker amplification. a,** Scheme of the DNA linker amplification. The DNA template strand is composed of two functional parts which are complementary to the primer (purple) and linker sequence (dark green). In addition, the strand contains the recognition part of the nickase to induce the linker amplification by a primer DNA without consuming it. **b, c,** The length of the DNA linker sequence was varied to analyse the constant linker production at a fixed amount of linker templates and primer DNA. The 40 nT linker production results in an inhibition of the polymerisation after 20 minutes. This is probably due to the strong interaction of linker and template strands, which prevents the replacement reaction of the linker. In contrast, a constant production of the a 20 nT linker was monitored over 4 hours of polymerisation. Here, 200 nM of prebuilt DNA-linker were added to 100 nM of converter to exclude the negative feedback of the DNA-linker.

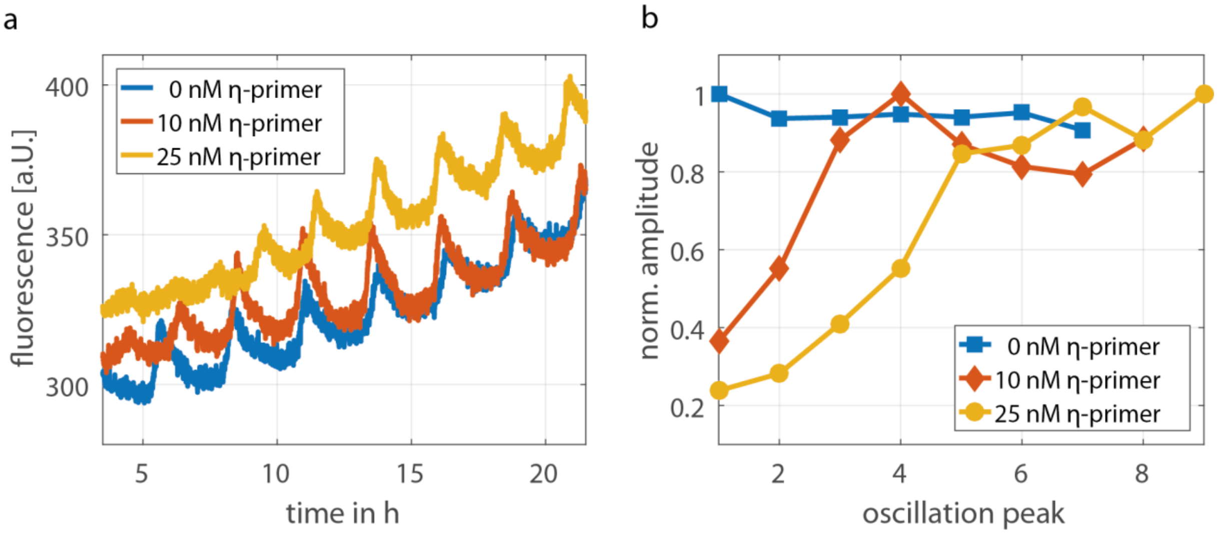


**Supplementary Fig. 6. Linker amplification primed by η strands. a,** Different concentrations of η-primed linker templates were added to the oscillatory network. Already 10 nM templates are sufficient to interfere with the oscillating network. **b,** The amplitudes of the different peaks were determined and plotted in an ascending order. A damping of the oscillations caused by the template can be seen. However, after around five cycles the system recovers, resulting in sustained oscillations.

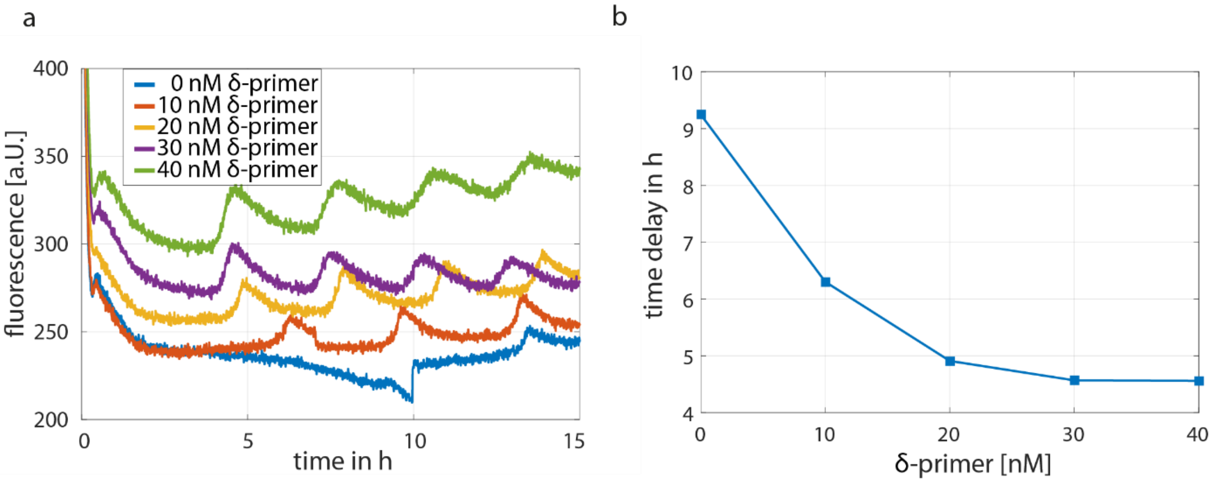


**Supplementary Fig. 7. Linker amplification primed by δ strands. a,** The oscillatory reaction network was observed for different concentrations of the of the δ-primed linker templates. The δ-templates reduce the lag-phase successively, leading to more robust oscillations. **b,** The lag-times between the first and second pulse were determined and plotted for the different template concentrations.

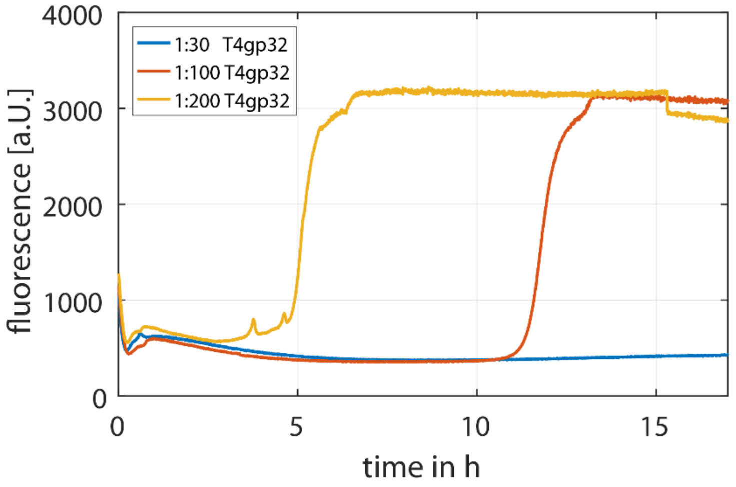


**Supplementary Fig. 8. Parasitic side-reaction.** The ab initio amplification of the Polymerase and Nickase leads to a random amplification of DNA sequences which interferes with the predator-prey reaction. The protein T4gp32 (stock concentration: 1 mg/ml) is used to prevent this reaction. It is a single-stranded DNA binding protein and used in PCR reactions to decrease the non-specific amplification. It was used at 33 µg/ml concentration, since higher concentration can lead to nonspecific interactions between the colloidal docking DNA.

```
% MATLAB code to generate Plot in Fig. 2c

%%%%%%%%%%%%%%%%%%%%%%%%%%%%%%%%%%%%%%%%%%%%%%%%%%%%%%%%%%%%%%%%%%%%%%%%%
close all;clear all

% Parameters

b     = 8.7*10^-2;
nb1   = 1*10^-3; nb5 = 5*10^-3; nb10 = 10*10^-3;
nb25  = 25*10^-3; nb50 = 50*10^-3;
k1    = 3.3*10^-2;
k2    = 2*10^-2;
k3    = 0.08*10^-3;
g     = 100*10^-3; B= 50*10^-3;
n(1)  = 10*10^-3;
p1(1) = 0;p5(1) = 0; p10(1) = 0; p25(1) = 0 ; p50(1) = 0;
time  = 7200;
t     = [1:time]./60

% Calculation

for i=2:time
n(i)  = n(i-1)   + g*n(i-1)*k1/(1+b*g*n(i-1)) ;
p1(i) = p1(i-1)  + B*p1(i-1)*k2/(1+b*B*p1(i-1))  + nb1*n(i-1)*k3 ;
p5(i) = p5(i-1)  + B*p5(i-1)*k2/(1+b*B*p5(i-1))  + nb5*n(i-1)*k3 ;
p10(i)= p10(i-1) + B*p10(i-1)*k2/(1+B*g*p10(i-1))+ nb10*n(i-1)*k3 ;
p25(i)= p25(i-1) + B*p25(i-1)*k2/(1+B*g*p25(i-1))+ nb25*n(i-1)*k3 ;
p50(i)= p50(i-1) + B*p50(i-1)*k2/(1+B*g*p50(i-1))+ nb50*n(i-1)*k3 ;
end

% Plot

plot(t,n)
ylim([0 1])
xlim([0 120])
hold on
plot(t,p1)
plot(t,p5)
plot(t,p10)
plot(t,p25)
plot(t,p50)
```
